# The SURF (Italian observational study for renal insufficiency evaluation in liver transplant recipients): a post-hoc between-sex analysis

**DOI:** 10.1186/s12882-019-1656-8

**Published:** 2019-12-23

**Authors:** Delia Colombo, Alessandro Zullo, Lucia Simoni, Emanuela Zagni, on behalf of the SURF Study Group, Stefano Fagiuoli, Paolo De Simone, Donato Donati, Mauro Salizzoni, Paolo Angeli, Patrizia Burra, Umberto Cillo, Pierluigi Toniutto, Massimo Rossi, Giovanni Vennarecci, Luciano De Carlis, Francesca Donato, Matteo Cescon, Alfredo Di Leo, Giovanni Giuseppe Di Costanzo, Alfonso Avolio

**Affiliations:** 1 Novartis Farma S.p.A, Largo Umberto Boccioni, 21040 Origgio, VA Italy; 2 MediNeos Observational Research, Modena, Italy

**Keywords:** Sex differences, Gender, Renal insufficiency, Liver transplant, Calcineurin inhibitors, Meta-analysis

## Abstract

**Background:**

Female sex has been reported as an independent predictor of severe post-liver transplantation (LT) chronic kidney disease. We performed a by sex post-hoc analysis of the SURF study, that investigated the prevalence of renal impairment following LT, aimed at exploring possible differences between sexes in the prevalence and course of post-LT renal damage.

**Methods:**

All patients enrolled in the SURF study were considered evaluable for this sex-based analysis, whose primary objective was to evaluate by sex the proportion of patients with estimated Glomerular Filtration Rate (eGFR) < 60 ml/min/1.73m^2^ at inclusion and follow-up visit.

**Results:**

Seven hundred thirty-eight patients were included in our analysis, 76% males. The proportion of patients with eGFR < 60 mL/min/1.73 m^2^ was significantly higher in females at initial study visit (33.3 vs 22.8%; *p* = 0.005), but also before, at time of transplantation (22.9 vs 14.7%; *p* = 0.0159), as analyzed retrospectively. At follow-up, such proportion increased more in males than in females (33.9 vs 26.0%, *p* = 0.04). Mean eGFR values decreased over the study in both sexes, with no significant differences. Statistically significant M/F differences in patient distribution by O’Riordan eGFR levels were observed at time of transplant and study initial visit (*p* = 0.0005 and 0.0299 respectively), but not at follow-up.

**Conclusions:**

Though the limitation of being performed post-hoc, this analysis suggests potential sex differences in the prevalence of renal impairment before and after LT, encouraging further clinical research to explore such differences more in depth.

## Background

Liver transplantation is the only available treatment option for several life-threatening liver conditions, including end-stage liver disease, acute fulminant hepatic failure, and hepatocellular carcinoma. Thanks to advanced surgical techniques and the progress achieved in perioperative management and immunosuppressive therapy, outcomes are currently satisfactory in terms of both short-term graft and patient survival. The overall 1-year survival rates after a liver transplant (LT) are 85% [1]. However, long-term outcomes of liver transplant recipients are not as satisfactory, given the high complication rate of long-term immunosuppressive therapy, including infections, malignancies and renal failure [2]. The overall 5- and 10-year survival rates after LT drop to 68, and 50% respectively [1]. Renal insufficiency, together with older age and diabetes, has shown to identify patients at highest risk of poor survival, and actually renal failure becomes a major cause of death with longer follow-up. A recent multicentre study has shown that the inflection point for increasing frequency occurs at the sixth postoperative year [1]. Furthermore, estimates suggest that around 20% of patients with chronic liver failure develop renal dysfunction [3], even before transplantation [4]. Elevated pre-operative serum creatinine levels are associated with increased risk of requirement for post-operative dialysis [5, 6], and those not receiving renal replacement therapy may be at greater risk of early graft failure than those receiving renal replacement therapy. A low threshold for instituting renal replacement therapy may therefore be beneficial [4, 7, 8]. The Kidney Disease Outcomes Quality Initiative (K/DOQI) guidelines recommend glomerular filtration rate (GFR) evaluation as the best global index of renal function, and state that it should be estimated based on formulas that consider the serum creatinine level and at least some of the following variables: age, sex, race and body surface [9]. An eGFR below 60 mL/min/1.73m^2^ is considered an index of renal damage [10, 11], and a recent systematic review of the methodology used in studies reporting chronic kidney disease prevalence reports that actually an eGFR < 60 mL/min/1.73m^2^ was used to define Chronic Kidney Disease (CKD) in 92% of studies [12]. Data from the literature show that patients undergone LT who have a GFR < 60 mL/min/1.73m^2^ 3 months following transplant are at high risk of developing chronic kidney disease [11, 13].

The SURF (Italian Observational Study for Renal Insufficiency Evaluation in Liver Transplant Recipients) was conducted from 2012 to 2014, with the aim of assessing the prevalence of reduced estimated Glomerular Filtration Rate (eGFR < 60 ml/min/1.73m^2^) in subjects underwent to primary orthotopic liver transplantation from 6 months to 5 years before entering the study. The SURF study showed an eGFR decline over time, particularly rapid in progression over the first year from transplant. This was hypothesized to be the result of the nephrotoxicity of CNIs, which were the most common therapeutic approach in the clinical practice.

Women represent a particular group of patients with chronic liver disease, not only given to a different body mass index, and different aetiologies of liver disease, and accessibility to transplantation, but also due to hormonal factors [14]. Nevertheless, sex is scarcely taken into account when indications, risk factors, and outcomes concerning LT are evaluated. In particular, regarding renal impairment, female sex has been reported as an independent predictor of severe post-LT chronic kidney disease [15]. The MetaGem project – i.e. the by sex analysis of the data form observational studies conducted between 2002 and 2013 – was started by Novartis Italy in 2013 [16]. These studies covered many different clinical areas, including autoimmune diseases, such as psoriasis and psoriatic arthritis, liver and kidney transplants, hepatitis B, and central nervous system diseases, including Parkinson and Alzheimer. Through post-hoc analyses and meta-analyses, the MetaGeM project is aimed at analysing and describing therapeutic approaches, clinical outcomes, and safety data by sex, to explore possible differences which may be useful in addressing future clinical research or specifically tailoring therapeutic approaches.

Within the overall MetaGeM project, we here report a post-hoc analysis of the SURF study data performed by sex, with the aim of exploring if there are differences between sexes in the prevalence and course of renal damage in LT recipients.

## Methods

SURF was an observational, multicentre, Italian, two phases study, one cross-sectional followed by a longitudinal phase. Male or female patients undergone first liver transplant in the previous 5 months to 5 years were enrolled in 15 Italian centres, provided they were aged ≥18 years at the time of transplantation, and were without combined multiple-organ transplant and not included in any experimental clinical trial at inclusion visit.

The SURF included just two visits, an enrolment visit, performed according to the routine clinical practice, when the retrospective data from inclusion to 30 days before LT were collected, and a follow-up visit approximately 12 months (± 3 months) after the inclusion visit, again performed as usual clinical practice. The type, dosage and duration of immunosuppressive therapy, and of all other medical treatments were according to clinical practice.

The primary endpoint of SURF study was the assessment of the proportion of patients with eGFR < 60 ml/min/1.73 m^2^ at inclusion and at the 12-month follow-up visit.

All patients enrolled in both the cross-sectional and longitudinal phases of the SURF study were considered evaluable for this sex-based analysis whose primary objective was to evaluate the proportion of patients with eGFR< 60 ml/min/1.73m^2^ at inclusion and follow-up visit by sex.

The secondary objectives were the following, evaluated on the longitudinal population and stratified by sex: (i) to describe eGFR distribution at inclusion and follow-up visit; (ii) to describe eGFR change (slope) from transplant to inclusion visit and from inclusion to follow-up visit (12-month observation period); (iii) to describe patient distribution by eGFR, proteinuria and slope category at inclusion and follow-up visit; (iv) to describe immunosuppressive therapy administered at inclusion visit and (v) to describe the therapeutic approach during the 12-month observation period based on eGFR status (eGFR < 60 ml/min/1.73m^2^ and eGFR ≥60 ml/min/1.73m^2^ at the inclusion visit).

As primary objective, the eGFR was computed according to the MDRD-4 formula [17, 18]: eGFR = 186 x (creatinine) ^-1.154^ x (age) ^-0.203^ x (1.212 if black) x (0.742 if female) where age is expressed in years and creatinine in mg/dL. If creatinine was reported in μmol/L, it was converted in mg/dL by means of conversion factor 0.0114, as follows: mg/dL =0.0114 x μmol/L. The prevalence was calculated as the ratio between patients with eGFR lower than 60 ml/min/1.73 m^2^ and the total number of evaluable patients.

As regards secondary objectives, eGFR distribution at inclusion visit was described using mean, standard deviation, quartiles, minimum and maximum. Furthermore, eGFR values were classified in 5 levels according to O’Riordan [19]: level 1 (eGFR ≥90 ml/min/1.73 m^2^), level 2 (eGFR between 60 and 89 ml/min/1.73 m^2^), level 3 (eGFR between 30 and 59 ml/min/1.73 m^2^), level 4 (eGFR between 15 and 29 ml/min/1.73 m^2^) and level 5 (eGFR < 15 ml/min/1.73 m^2^). Absolute and relative frequencies were provided.

The eGFR annual average change (slope) from transplant to inclusion visit was calculated as follows: (eGFR at time t2 – eGFR at time t1) / [(t2-t1)/365.25] where t1 = date of creatinine assessment at transplant, and t2 = date of creatinine assessment at inclusion visit. eGFR change per patient was described using mean, standard deviation, quartiles, minimum and maximum. eGFR slope was described overall and by time from transplant.

The GFR level, its variation (slope) and proteinuria are important elements that, if combined, could provide an early picture of the renal function condition. From the combination of eGFR, slope and proteinuria and according to literature data, the Advisory Board of the SURF study identified different categories of renal dysfunction, as showed in Table 1 [1, 20–24]. The distribution of LT patients’ according to these categories was provided too.

**Table 1:** Combination of eGFR, proteinuria and slope values defining the SURF study categories

Category	eGFR	Proteinuria	Annual slope^a^
1	≥ 90 ml/min	NO/NA	Any
2	≥ 90 ml/min	YES	Any
	60–89 ml/min	NO/NA	Decrease ≤4 ml/min or any increase
3	60–89 ml/min	NO/NA	Decrease > 4 ml/min
	60–89 ml/min	YES	Decrease ≤4 ml/min or any increase
	30–59 ml/min	NO/NA	Decrease ≤4 ml/min or any increase
4	60–89 ml/min	YES	Decrease > 4 ml/min
	30–59 ml/min	NO/NA	Decrease > 4 ml/min
	30–59 ml/min	YES	Decrease ≤4 ml/min or any increase
5	30–59 ml/min	YES	Decrease > 4 ml/min
	< 30 ml/min	NO/NA	Decrease ≤4 ml/min or any increase
6	< 30 ml/min	NO/NA	Decrease > 4 ml/min
	< 30 ml/min	YES	Any

Categories were identified by the Advisory Board of the SURF study

*NA* Not available

^a^Annual slope is intended as annual average variation of eGFR obtained over a period ≥5 months

To describe the therapeutic approach according to eGFR values during the 12-month observational period, the proportion of patients who changed therapy during observation period was described by eGFR classes (i.e. eGFR< 60 ml/min/1.73 m^2^ vs eGFR ≥60 ml/min/1.73 m^2^) at inclusion visit.

Patients with missing data for one or more variables were not excluded from the analyses, they simply were not evaluated for that variable(s). Comparisons between males and females were performed by Student *t*-test, Wilcoxon-Mann-Whitney test, χ^2^ test and Fisher exact test if appropriate. The accepted level of significance was set to alpha = 0.05.

The analyses were performed using SAS v.9.2 and Enterprise Guide v.4.3.

## Results

Out of 1029 enrolled patients in the SURF study, 1002 (97.4%) met eligibility criteria for the cross-sectional phase; out of those patients, 753 entered in the longitudinal phase, of whom 738 were evaluable and included in the analysis. Out of these 738 patients, 561 (76.0%) were males. Demographic and baseline characteristics, and patients’ history of liver disease and liver transplantation are summarized in Table 2. The mean ± SD age at inclusion was 56.2 ± 8.7 in men vs 53.2 ± 12.2 years in women (T-test *p*-value = 0.00291) and the mean ± SD age at transplant was 53.8 ± 8.5 in men vs 50.8 ± 12.2 years in women (*p*-value (T-test) = 0.00283). The proportion of HCV-positivity was significantly higher in males (47.4% vs 28.8% in females; *p*-value (χ^2^ test) < .0001).

**Table 2:** Demographic and baseline characteristics, and history of LT

	Females	Males	Total
	(*n* = 177)	(*n* = 561)	(*n* = 738)
Age (years) at LT, mean (SD)	50.8 (12.2)	53.8 (8.5)	53.1 (9.6)
Age (years) at inclusion visit, mean (SD)	53.2 (12.2)	56.2 (8.5)	55.5 (9.7)
BMI 6 months after LT, mean (SD)	24.0 (4.1)	25.0 (3.1)	24.8 (3.4)
BMI at inclusion visit, mean (SD)	25.2 (4.5)	26.1 (3.3)	25.8 (3.7)
BMI at follow-up visit, mean (SD)	25.7 (4.9)	26.4 (3.5)	26.2 (3.9)
Time (months) from liver transplant to inclusion visit, mean (SD)	29.1 (16.1)	28.9 (16.3)	29.0 (16.2)
Time (months) from liver transplant to inclusion visit by class			
[5 months; 1 year)	31 (17.5%)	97 (17.3%)	128 (17.3%)
[1 year; 2 years)	46 (26.0%)	158 (28.2%)	204 (27.6%)
[2 years; 3 years)	38 (21.5%)	105 (18.7%)	143 (19.4%)
[3 years; 4 years)	31 (17.5%)	108 (19.3%)	139 (18.8%)
[4 years; 5.5 years]	31 (17.5%)	93 (16.6%)	124 (16.8%)
MELD score at transplant, mean (SD)	18.2 (8.4)	17.47 (7.1)	17.7 (7.4)
MELD score at transplant by class, N (%)			
Low MELD (<=14)	54 (36.2%)	196 (41.4%)	250 (40.2%)
Intermediate MELD (15–24)	65 (43.6%)	208 (44.0%)	273 (43.9%)
High MELD (> = 25)	30 (20.1%)	69 (14.6%)	99 (15.9%)
NA	13	38	51
Not Recorded	15	50	65
Main conditions leading to LT (> 10%), N (%)			
Hepatocellular carcinoma in cirrhotic liver + cirrhosis, HCV+	27 (15.3%)	143 (25.5%)	170 (23.0%)
Hepatocellular carcinoma in cirrhotic liver + cirrhosis, HCV-	15 (8.5%)	110 (19.6%)	125 (17.0%)
Cirrhosis, HCV+	23 (13.0%)	120 (21.4%)	143 (19.4%)
Cirrhosis, HCV-	70 (39.6%)	149 (26.6%)	219 (29.7%)
HCV+ and HBV+ patients having hepatocellular carcinoma in hepatic liver or cirrhosis as disease leading to liver transplantation			
HBV+	34 (19.2%)	137 (24.4%)	171 (23.2%)
HCV+	51 (28.8%)	266 (47.4%)	317 (43.0%)

*LT* liver transplantation, *BMI* body mass index, *MELD* Model for End-stage Liver Disease, *HBV* hepatitis B virus, *HCV* hepatitis C virus

Eight patients had chronic liver rejection ongoing at inclusion visit (4 males and 4 females), while one or more acute liver rejections from transplant to inclusion visit had been experienced by 129 patients (12.8%; 16.6% of females and 11.7% of males; *p*-value (χ^2^ test) males vs females = 0.04767).

Overall, 187 patients (25.3%) had an eGFR < 60 mL/min/1.73 m^2^ at the initial study visit, representing 22.8% of the male population and 33.3% of females (*p*-value (χ^2^ test) males vs females = 0.0050), while at the follow-up visit the proportion increased overall to 27.9% (*n* = 206), and the increase was greater in males (26.0%) than in females (33.9%) (*p*-value (χ^2^ test) males vs females = 0.04176). Retrospectively analysing eGFR values at the time of transplantation, the overall percentage of patients below 60 mL/min/1.73 m^2^ was lower (16.7%), and already slightly significantly higher among females (22.9%) than males (14.7%) (*p*-value (χ^2^ test) males vs females = 0.0159).

Mean (SD) eGFR values decreased from a mean value of 94.5 (37.3) ml/min/1.73 m^2^ at transplant to 76.2 (25.5) at inclusion visit and to 74.6 (25.4) at follow-up visit, with no significant differences between males and females. Patient distribution according to O’Riordan levels of eGFR at each time point are summarized in Table 3. Statistically significant male/female differences were observed at the time of transplant and at the study initial visit (Fisher exact test *p*-values 0.0005 and 0.0299 respectively), but not at follow-up.

**Table 3:** Patient distribution according to O′ Riordan et al. (2006) levels of eGFR (MDRD-4)

	Males	Females	Total	*p*-value
At transplant (ml/min/1.73m^2^)				
Level 1: eGFR ≥90	288 (57.3%)	65 (41.4%)	353 (53.5%)	0.0005^a^
Level 2: eGFR 60–89	141 (28.0%)	56 (35.7%)	197 (29.8%)	
Level 3: eGFR 30–59	67 (13.3%)	29 (18.5%)	96 (14.5%)	
Level 4: eGFR 15–29	7 (1.4%)	4 (2.5%)	11 (1.7%)	
Level 5: eGFR < 15	0 (0.0%)	3 (1.9%)	3 (0.5%)	
Not Recorded	58	20	78	
At inclusion visit (ml/min/1.73m^2^)				
Level 1: eGFR ≥90	156 (27.8%)	48 (27.1%)	204 (27.6%)	0.0299^a^
Level 2: eGFR 60–89	277 (49.4%)	70 (39.6%)	347 (47.0%)	
Level 3: eGFR 30–59	117 (20.9%)	57 (32.2%)	174 (23.6%)	
Level 4: eGFR 15–29	5 (0.9%)	1 (0.6%)	6 (0.8%)	
Level 5: eGFR < 15	6 (1.1%)	1 (0.6%)	7 (1.0%)	
At follow-up visit (ml/min/1.73m^2^)				
Level 1: eGFR ≥90	137 (24.4%)	39 (22.0%)	176 (23.9%)	0.25903^b^
Level 2: eGFR 60–89	278 (49.6%)	78 (44.1%)	356 (48.2%)	
Level 3: eGFR 30–59	133 (23.7%)	54 (30.5%)	187 (25.3%)	
Level 4: eGFR 15–29	8 (1.4%)	5 (2.8%)	13 (1.8%)	
Level 5: eGFR < 15	5 (0.9%)	1 (0.6%)	6 (0.8%)	

*eGFR* estimated Glomerular Filtration Rate, *MDRD-4* 4 variable Modification of Diet in Renal Disease study equation

^a^Fisher exact test; ^b^χ^2^ test

Annual average eGFR change (slope) from transplant to inclusion visit was much higher in the subgroup of patients undergone liver transplantation 6–12 months before SURF inclusion visit (Table 4), and almost double in females compared to males. The annual average change in eGFR, as assessed during each year from transplant, was also higher during the first post-transplant year (mean − 16.4 ml/min/1.73 m^2^) than in the following years, with a greater decline in men (mean − 18.0) than in women (mean − 11.2) that did not achieve statistical significance. The median difference between sexes was significant only at the fourth post-transplant year, when females improved, and men slightly declined. The median [25th percentile; 75th percentile] was + 1.4 ml/min/1.73 m2 [− 2.4;13.3] in females and − 1.8 [− 8.3;6.1] in males, *p*-value (Wilcoxon-Mann-Whitney test) = 0.0024. Proteinuria was present in 20.9% of patients at inclusion and in 23.1% at follow-up, with no between sexes difference.

**Table 4:** eGFR (MDRD-4) annual average change (slope) overall and by time from transplant to inclusion visit

		eGFR annual average change (slope) from transplant to inclusion visit (ml/min/1.73m^2^/year)						*p*-value
Time elapsed from transplant to inclusion visit		N	Mean	SD	25th percentile	Median	75th percentile	
[5 months; 1 year)	Females	30	−41.6	95.1	−62.0	−24.6	9.2	0.29355*
	Males	92	−22.3	50.0	−54.0	−14.8	9.6	
	Total	122	−27.1	64.2	−56.1	−16.9	9.5	
[1 year; 2 years)	Females	41	− 5.5	27.3	−23.2	−6.9	3.6	0.1478**
	Males	140	−14.8	23.7	−27.6	−11.2	−0.5	
	Total	181	−12.6	24.8	−25.9	−9.7	−0.3	
[2 years; 3 years)	Females	33	−6.8	17.7	−12.7	−5.1	−0.2	0.54312*
	Males	95	−8.7	14.7	−17.0	−7.6	0.2	
	Total	128	−8.3	15.5	−16.2	−7.1	−0.2	
[3 years; 4 years)	Females	28	−4.6	8.1	−10.3	−5.9	−1.0	0.40157*
	Males	95	−6.3	9.5	−11.1	−5.3	0.2	
	Total	123	−5.9	9.2	−10.8	−5.3	−0.2	
[4 years; 5.5 years]	Females	25	−3.7	11.1	−9.0	−3.7	2.6	0.94751*
	Males	81	−3.9	7.8	−10.5	−3.5	1.4	
	Total	106	−3.8	8.6	−10.3	−3.6	2.6	
Total evaluable patients	Females	157	−12.2	46.6	−16.7	−5.9	2.6	0.87949*
	Males	503	−11.6	26.8	−19.6	−6.7	0.9	
	Total	660	−11.8	32.6	−19.0	−6.5	1.2	

*eGFR* estimated Glomerular Filtration Rate

*SD* Standard deviation

*Two-sample T-test *p*-value

** Wilcoxon-Mann-Whitney test *p*-value

**Fig. 1:**
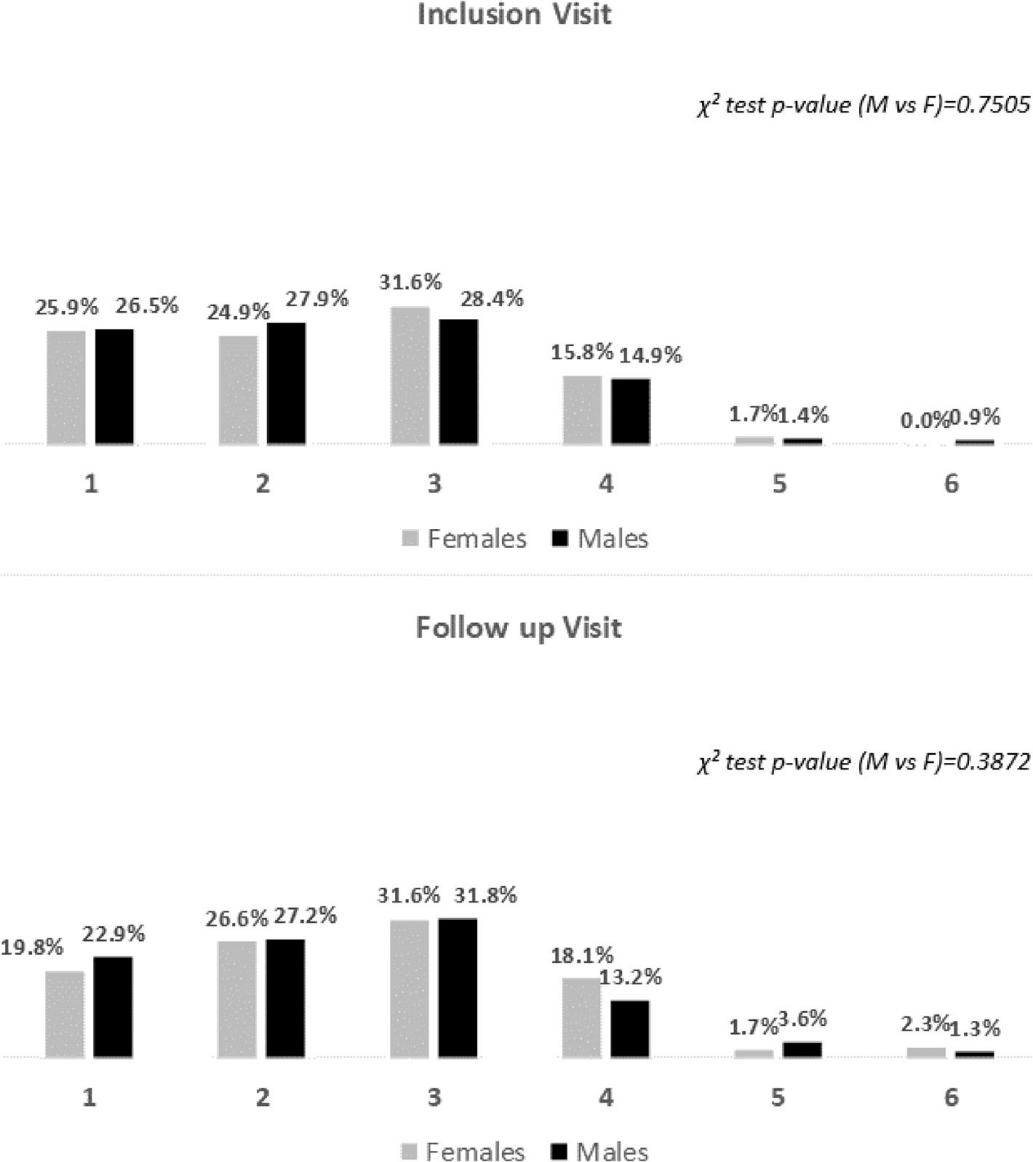
eGFR, proteinuria and slope categories at inclusion and at follow-up visit by sex

Coming to the eGFR, proteinuria and slope combined categories, there was a little shift from the first two classes to the higher ones from inclusion to follow-up visit, but with no significant differences between sexes (see Fig. 1).

Immunosuppressive therapy, as registered at inclusion visit, included CalciNeurin Inhibitors (CNI) in more than 90% of patients (74% tacrolimus, 18% cyclosporine): in 50.8% as monotherapy, and in 38.6% in combination with mycophenolate or everolimus, the rest with other medications. At least one change in immunotherapy in terms of actives was reported in 46.9% of patients from transplant to inclusion visit with no differences between sexes (*p*-value (χ^2^ test) males vs females = 0.73182), and in 16.3% from inclusion to follow-up visit, with no differences between eGFR levels (*p*-value (χ^2^ test) eGFR < 60 ml/min/1.73 m^2^vs eGFR ≥60 ml/min/1.73 m^2^ = 0.19953) nor sexes (*p*-value (χ^2^ test) eGFR < 60 vs eGFR ≥60 in male population = 0.07929; *p*-value (χ^2^ test) eGFR < 60 vs eGFR ≥60 in female population = 1.000).

## Discussion

The SURF study, was conducted from 2012 to 2014, with the aim of assessing the prevalence, in subjects underwent to primary orthotopic liver transplantation, of eGFR < 60 ml/min/1.73 m^2^, which is a recognized index of renal damage and an indicator of poor post-transplant prognosis. The results have been first analysed in the overall study population. Then, given the growing interest in sex medicine and the recognised need to be mindful of sex differences in designing clinical protocols and treatment pathways to improve outcomes in LT [16, 25–27], a post-hoc by-sex analysis of the SURF patients participating in the longitudinal follow-up phase was performed.

The first relevant result is that three quarter of the transplanted patients in the participating centres were males. This is not surprising, given that epidemiological data report that men have higher rates of liver cancer than women, and liver cancer is a common cause of LT. It has been postulated that oestrogens play a protective role in women with chronic liver disease: experimentally, oestrogens have been shown to exert a strong suppressive role on fibrosis in a rat model [28, 29], and in the clinical setting, menopause has been associated with higher degrees of fibrosis [30, 31]. Another issue, may be the access to LT among women. It has been recently reported that females comprised 35% of transplant recipients in 2013 [32], their number constantly declined since 2002 [27]. Data suggest that this proportion of women is also less likely to undergo LT once listed and have a greater probability of dying or becoming too sick to undergo liver transplantation compared to men [33].

Except from the size, male and female populations in our study were rather homogeneous for demographic and clinical baseline characteristics, however a significantly higher proportion of HCV-positive subjects was registered among males. Once again, this is consistent with previous data indicating that hepatitis B and C are more common in males [34–36]. A National Health and Nutrition Examination Survey (NHANES) conducted between 2003 and 2010 found men to be significantly more likely to be chronically infected with hepatitis C virus than women [37]. Moreover, the progression of fibrosis in patients with chronic HCV is twice as rapid in men versus women [38, 39], and this may further justify the greater prevalence of men undergoing LT.

Following LT, eGFR values, according to the literature, are highly variable and dependent from time elapsed from transplant: in the TRY study [40], the percentage of patients with eGFR < 60 ml/min/1.73 m^2^ ranged from 48% after 1 month to 58% after 5 years from transplant. According to Kim et al. 2010, chronic kidney disease is observed in 10 to 60% of patients up to 5 years after transplantation. In our overall population, the proportion of patients with eGFR < 60 ml/min/1.73 m^2^ at enrolment (25.3%) fell within the lower range of proportions reported in the literature. A possible reason for this may be a potential selection of patients with good renal prognosis: namely, patients who received combined multiple-organ transplant or liver re-transplant were not included in the study, as per exclusion criteria.

Previous estimates suggest that around 20% of patients with chronic liver failure develop renal dysfunction even before LT [3], and this is associated with reduced survival in patients undergoing both elective and urgent orthotopic LT [7]. At the time of LT, in our population, the prevalence of renal damage, as assessed by an eGFR < 60 mL/min/1.73 m^2^, was 16.67%, which is only slightly lower than literature data. The increase in the prevalence of impaired renal filtration observed at the time of initial study visit (+ 52% vs LT-time) and further during the 1-year follow-up (+ 67% vs LT-time), confirms that renal dysfunction had developed in many more patients after transplantation. Consistently the mean eGFR values progressively decreased from transplant time to follow-up. The slope of the eGFR decline, as well as the annual average change, were much higher during the first post-transplant year, suggesting a more rapid worsening of renal function early after LT. Acute renal failure (ARF) is a serious and frequent clinical problem associated with liver LT: the incidence of post-LT ARF has been reported to range between 17 and 95% in different studies and may also be associated with LT surgery [41–44].

The proportion of patients with an impaired GFR was significantly higher in females at both time of transplantation and study inclusion visit. This suggests that our female patients arrived at LT in worse renal conditions compared to males, as confirmed also by patients’ distribution among O’Riordan eGFR levels, that shows a highly significant sex-difference at the time of LT, once more indicating less renal impairment in men. This observation seems to confirm the hypothesis by Moylan et al. [31] that sex disparities still exist in access to LT and women often arrive at transplantation in worse clinical conditions, sometimes even too ill to actually receive a transplant. On the other hand, it has also to be considered that HCC patients are typically transplanted with MELD exception; thus, they do not need to accumulate renal dysfunction in order to access to transplant, as creatinine is part of the MELD calculation. This too may explain why males had less renal dysfunction at transplant than females.

When looking at the annual average filtration decline, interestingly it was lower in women, not reaching statistical significance in first post-LT year but becoming significant at the fourth year after transplantation, when females had even improved and men further slightly worsened. This is interesting and somewhat surprising, but might be due to the generally greater muscle mass of males and to the fact that the use of eGFR has major limitations in cirrhotic patients. During post-LT course, significantly more females experienced one or more acute liver rejections from transplant to inclusion visit.

Renal failure remains a major cause of late mortality after LT [1]. A clear link is recognised between renal failure and long-term immunosuppressive therapy, and prolonged use of CNI, in particular, has been associated with chronic nephrotoxicity after all types of transplantation, as well as during treatment of autoimmune disease. Almost all transplanted patients in the SURF were on CNI-based immunosuppressive therapy, mainly with tacrolimus, either alone or in combination, and this may have significantly contributed to renal impairment following LT. Considering the more benign trend in eGFR levels in females compared to males, we wonder whether women may be less sensitive to the CNI-associated renal damage. No significant differences in the treatment approaches have been found, so that we may exclude that the differences observed in the filtration rate trend may be due to different therapeutic behaviours. Recently, it has been suggested that delayed introduction of CNI following LT, and personalization of immunosuppression by identifying those who may gain maximum benefit from CNI-avoidance or minimization may, theoretically, help decrease the negative impact of CNI on renal function [45–48].

Our study has the limitation of a post-hoc by-sex analysis, which was not a specified objective of the original SURF study. Therefore, the sample size had not been calculated in order to detect sex differences in the study endpoints and male and female patients were not properly balanced. The observational design and the retrospective collection of LT data represent other limitations, in terms of data availability and homogeneity. These limitations in the study design make it difficult to draw definitive conclusions regarding sex and renal function outcome after LT.

## Conclusions

We think that the SURF study sheds some light on the prevalence of renal impairment before and after LT, and this post-hoc by-sex analysis suggests potential sex differences, encouraging additional clinical research to confirm and further explore such differences in the complex setting of LT. We strongly believe and have also shown in some recent works [49–52], that men and women differ with regard to severity and pathogenesis of diseases, healthcare needs and drug tolerability, and that a uniform approach may not always be the best choice for the patient. Also in the management of liver transplant recipients, sex-based approaches may be needed to optimize modifiable risk factors and improve post-transplant renal function.

## Data Availability

All data analysed during this study are included in this published article.

## Abbreviations

ARF: Acute renal failure
CKD: Chronic kidney disease
CNI: Calcineurin inhibitors
eGFR: estimated glomerular filtration rate
GFR: Glomerular filtration rate
HCV: Hepatitis C virus
K/DOQI: Kidney Disease Outcomes Quality Initiative
LT: Liver transplant
NHANES: National Health and Nutrition Examination Survey
